# Characterizing Social Determinants of Maternal and Child Health: A Qualitative Community Health Needs Assessment in Underserved Areas

**DOI:** 10.3390/healthcare11152224

**Published:** 2023-08-07

**Authors:** Sara Rizvi Jafree, Gulzar Shah, Rubeena Zakar, Anam Muzamill, Humna Ahsan, Syeda Khadija Burhan, Ambreen Javed, Rana Rubab Durrani

**Affiliations:** 1 Department of Sociology, Forman Christian College University, Zahoor Elahi Road, Lahore 54600, Pakistan; sarajafree@fccollege.edu.pk; 2 Department of Health Policy and Community Health Jiann-Ping Hsu College of Public Health, Georgia Southern University, Statesboro, GA 30460, USA; 3 Institute of Social and Cultural Studies, University of the Punjab, Lahore 54590, Pakistan; rubeena499@gmail.com; 4 Department of Mass Communications, Forman Christian College University, Lahore 54600, Pakistan; anammuzamill@fccollege.edu.pk; 5 Department of Economics, Forman Christian College University, Lahore 54600, Pakistan; humnaahsan@fccollege.edu.pk; 6 Department of Education, Forman Christian College University, Lahore 54600, Pakistan; khadijaburhan@fccollege.edu.pk; 7 Department of English, Forman Christian College University, Lahore 54600, Pakistan; ambreenjaved@fccollege.edu.pk; 8 Center for Language Development, Forman Christian College University, Lahore 54600, Pakistan; rubabdurrani@fccollege.edu.pk

**Keywords:** social determinants of health, environment, community needs assessment, maternal and child health

## Abstract

This study aimed to identify social determinants of maternal and child health (SDoH) in Pakistan. Using a qualitative study design, data were collected from community members in seven underserved areas of Lahore City, Pakistan. A total of 22 qualitative in-depth interviews and 10 focus group discussions (FGDs) were conducted. The participants included basic health unit healthcare staff, women of reproductive ages, male family members, mothers-in-law, and religious leaders. We found that maternal and child health is adversely affected by the following socioeconomic and environmental barriers: (i) poor housing quality and sanitation; (ii) inadequate food supply and safety; (iii) unsatisfactory public sector school services; (iv) a lack of safety and security; (v) scarce poverty alleviation efforts and loan schemes; (vi) unsatisfactory transport and internet services; and (vii) inadequate health services. The targets for maternal and child health in Pakistan cannot be met without close coordination between the primary health sector, local governance, and macro state structures, which collectively must monitor and improve housing adequacy, food security, public sector services (primary healthcare services, public schooling, public transport, and public internet access), overall safety, and poverty emergence.

## 1. Introduction

There are very few community-based studies that attempt to identify the socioeconomic and environmental challenges that act as barriers to maternal and child health (MCH) in Pakistan. In the absence of evidence-based program planning and a non-conducive community environment, even a well-functioning primary health center for MCH may not accomplish the desired MCH outcomes [1]. South Asian countries have made significant efforts to meet the World Health Organization’s Sustainable Development Goals for MCH [2], but some of these countries are challenged by rising populations living in communities that are disadvantaged by a lack of access to social determinants of health (SDoH) such as MCH-related literacy, prenatal and postnatal care access, access to healthy and sufficient calories, safe and uncrowded housing, and affordable transportation for MCH care, to name a few [3,4]. The urban slum populations living in large cities are a major concern, as urban zones may report primary healthcare services for maternal and child health, but access and optimal health outcomes for the poor are compromised due to inadequate housing and sanitation, poor or no access to MCH care, and inequitable access to other SDoH [5]. For instance, meeting maternal and child health targets in the developing world is highly dependent on access to improved water, sanitation, and hygiene for women and their families. Research shows that poor household construction materials for walls, floors, and ceilings, as well as inadequate access to electricity, water, and toilet facilities, are associated with diarrhea incidence in children [6]. Poor housing conditions can lead to adverse health problems, including infectious diseases, respiratory disorders, stress, and depression [7]. Low-income families living in rental houses with unaffordable rents are known to suffer from greater mental and physical health burdens, including a greater risk of being underweight, being hospitalized, facing depression, and also food insecurity [8].

In developing countries, the situation of MCH-related SDoH is deteriorating. Increasing population growth rates are worsening the situation of food insecurity, and the affordability of basic food items, in turn, negatively impacts maternal and child health [9]. To illustrate, a lack of adequate and nutritious food is known to be associated with miscarriages [10] and the low birth weight of newborns [11]. Poor access to clean water and sanitation increases the risk of child and maternal mortality [12,13]. Many women living in low-resource rural communities have to travel long distances on foot to fetch water and spend time, effort, and fuel in boiling it for safe drinking, which encroaches into their time for other health-seeking practices, such as the nurturing of children and self-care [14].

In a community-based study on the relationship between health and community environment, residents reported that safe places for children’s recreation, good quality public school services, and safe neighborhood walk trails are important factors in influencing maternal and child health [15]. Environmental hazards in the community, such as open gutters, open waste, and overall neighborhood poverty are known to be associated with higher infection rates and disease burdens in residents [16]. Another community assessment showed that greater health vulnerability existed in neighborhoods with inadequate housing, drainage, garbage disposal, or safety against crime and unfair rental schemes [17]. Household-level financial hardship may also have an immense impact on maternal and child health, with challenges such as undernutrition, low-health-seeking behaviors, low immunization coverage, and low expenditure on education [18]. The affordability of fresh fruits and vegetables in the neighborhood also affects the health of the mother, child, and the entire family [17]. Disadvantaged communities which have better access to loans [19] and insurance schemes [20] are known to have better outcomes for maternal and child health.

Research studies in the developing world show that, in communities, the accessibility and affordability of public transportation can be significant barriers to mobility, negatively impacting maternal and child health [21]. In conservative communities, cultural and family barriers prevent women from using public transport [22]. Communities with good internet access, on the other hand, facilitate families’ access to health information and telehealth, which, in turn, leads to good MCH [23]. Internet access can also be a source of information for income generation, small business opportunities, and social services [24].

The 18th amendment to the Constitution of Pakistan devolved health to the provincial governments in the hopes that there would be more region-specific planning for health. However, the lack of both a national vision and provincial efforts has landed Pakistan in a dismal state for MCH, with the third highest burden of maternal and child mortality in the world [25]. The lack of a comprehensive database and accurate data on MCH and a lack of efforts to collect data on the wide-ranging SoDoH, such as the complex environment and living conditions in urban cities, has contributed to ineffective planning to reduce maternal and child mortality [26].

Pakistan is also facing high rates of communicable disease burden due to inadequate living conditions and uncontrolled infection, with pregnancy-related health conditions and malnutrition comprising half of the burden [27]. The main SDoH remain health illiteracy, cultural barriers, and inadequate or low-quality services for mother and child [28]. Maternal and child deaths are preventable through better living environments, better quality services and reach, and support for removing cultural and financial barriers [29]. A recent assessment in Lahore, Pakistan, for an MCH intervention concluded that improvement was not possible without the collaboration of multi-dimensional stakeholders, such as local governance, central government, and the private sector [30]. A recent Urban Slum Profile from poor urban areas of Lahore and other cities revealed there are major shortages of health facilities and female health workforce in communities to serve women and children [31]. Additionally, there is low availability of schools, and the living environment is inadequate, with inferior housing.

In Pakistan, the thrust of public health interventions to address maternal and child health (MCH) issues have been on individual behavioral modification and direct provision of MCH services. The primary intervention has been the state-run Lady Health Worker Program, through which door-to-door services in the community are delivered to at least 60% of women of reproductive years in the country [32]. The Lady Health Worker (LHW) is also available in the local basic health unit for women to visit and gain services from; however, these services are limited to reproductive health.

The modern public health enterprise in the Public Health 3.0 era advocates for the adoption of the system’s approach in assessing broader upstream social, economic, and institutional influences, referred to as social determinants of health (SDoH), as well as shaping these SDoH through the Health in All Policies (HiAPs) approach to address downstream health outcomes and disparities in those outcomes [33]. The Public Health 3.0 and HIAP approaches advocate for approaching MCH challenges as attributable to a complex web of social disadvantages or advantages that are not distributed equally or equitably. The focus of existing research studies in Pakistan on challenges facing MCH is predominantly on individual-level factors, exploring correlations through quantitative factors, leaving a critical gap in actionable research evidence. This qualitative research aims to study the socioeconomic and environmental challenges in disadvantaged urban slum areas that present barriers to maternal and child health using a community-based needs assessment approach. The study findings yield critical evidence to inform policy aiming for holistic community development to meet maternal and child health targets. The central research question was: do any of the following community and neighborhood services influence the health of mothers and children through SDoH, including (i) housing quality and sanitation services; (ii) food and water safety; (iii) public sector school services for children; (iv) safety and security in the neighborhood; (v) poverty alleviation and loan schemes; and (vi) transport and internet services?

## 2. Materials and Methods

### 2.1. Research Design and Data

This study uses a qualitative research design based on data from 22 in-depth qualitative interviews and 10 focus group discussion participants, representing the public health workforce and community residents such as women of reproductive ages, mothers-in-law, and male members of the households. This research is part of the project titled “Evidence-based Intervention to Improve Social Policy for Maternal and Child Health using a Pre-Post Test Design”, funded by the Higher Education Commission of Pakistan through the National Research Program for Universities. This is a two-year project that comprises four streams: (1) collection of baseline data for health and social indicators of disadvantaged women of reproductive years and index development for maternal health and well-being; (2) implementation of a health and social literacy intervention for disadvantaged women of reproductive years; (3) pre- and post-intervention tests to identify the impact of the health and social literacy intervention; (4) teambuilding workshops for basic health unit (BHU) team, deployed for primary care in underserved communities; (5) a community needs assessment to identify the socioeconomic and environmental challenges facing disadvantaged women of reproductive years and preventing optimal maternal and child health outcomes. This study only reports the findings from the qualitative data from the community needs assessment. Ethics approval for this study was obtained from the [Blinded for Review], Institutional Review Board, reference code = IRB-257/04-2021. All participants of the study gave informed consent. The anonymity, confidentiality, and safety of participants were ensured.

The Community Tool Box was used for the assessment of communities [34], which is an adequate instrument used to assess underdeveloped regions and identify problems facing women and families. Focus group discussions were conducted using a qualitative guide. A semi-structured interview guide (see Supplementary Materials File S1) was prepared using the Community Tool Box, which asks participants to share information about how maternal and child health is influenced by the existing services and the following social determinants of MCH: (i) housing and sanitation, (ii) water and food security, (iii) public sector services, such as schools, healthcare, transport, and safety, (iv) the availability of loan, entrepreneurial, and poverty schemes, and (v) the availability of public transport and internet services. A pilot test took place in October 2022 to help develop prompts and finalize the interview guide.

### 2.2. Data Collection

The community needs assessment took place in seven underserved areas of Lahore city which have a functioning BHU, including (1) Bhasin; (2) Shahpur, Alama Iqbal Town; (3) Munir Garden; (4) Township Block 2, Sector C; (5) H.B.F.C Society; (6) Sundar Sharif, Raiwind Road; and (7) Jallo Park. More details about the data collection sites, including areas, addresses, and Google map locations, are summarised in Supplementary Materials File S2. The inclusion criterion was participants living in the community as permanent residents for at least the last year, whereas the exclusion criterion was temporary community residents who had not been residing in the region for the last year.

Both in-depth interviews (IDIs) and focus group discussions (FGDs) were conducted with the following community members (Table 1): (i) BHU team (4 FGDs); (ii) Lady Health Workers (LHWs) (4 IDIs); (iii) women of reproductive years (6 IDIs and 6 FGDs); (iv) male household members (5 IDIs); (v) mothers-in-law (3 IDIs); and (vi) local community religious leaders (4 IDIs). Focus group discussions were conducted at neutral venues in the communities, and participants were identified by the community liaisons for the project. The data from both IDIs and FGDs were collected by six of the authors of this paper (SRJ, AM, HA, SKB, AJ, and RRD) and their research team across three weeks, between January to February 2023. Some select images showing living conditions of the people are presented in Supplementary Materials File S3. The BHU teams were requested for support to arrange interviews with women clients, their family members, and local religious leaders.

### 2.3. Data Analysis

Data were audio-recorded and transcribed by the researchers. The first and fourth authors then verified the transcribed data. Thematic analysis steps, recommended by Nowell et al. (2017) were followed to ensure the trustworthiness of thematic coding [35]. Three of the authors (SRH, AM, and HA) independently reviewed the data and generated codes and then had meetings to review and confirm theme generation. Following the grounded theory approach, the emergent coding technique was used to determine themes, as emergent coding allowed the researchers to generate themes after multiple rounds of reading the transcribed data. The process was comprised of generating the initial codes, generating and vetting the themes and sub-themes with team members, and finalizing the theme names and definitions. The final themes were shared with BHU staff and five community participants (women of reproductive years) who confirmed the findings.

## 3. Results

All the LHWs (*n* = 4) and other BHU team members (*n* = 10) in our study were government employees between the ages of 35 and 50 years. There were 31 women of reproductive years sampled between the ages of 18 and 45 years, all of whom had at least one child. Three mothers-in-law were sampled between the ages of 45 years and 60 years. There were five male household members sampled between the ages of 20 and 72 years, all of whom had at least one child. Four Muslim religious leaders were sampled, who were between the years of 45 and 72 years, all of whom were currently leading Friday sermons and prayers in the local community mosque.

The following six themes emerged in the thematic analysis under two broad headings, as discussed below: 1. Socioeconomic challenges: (i) scarce poverty alleviation and loan schemes; (ii) inadequate food supply and safety; (iii) unsatisfactory public sector services (schools and health services). 2. Environmental challenges: (iv) poor housing quality and sanitation services and rent burden; (v) lack of safety and security; (vi) unsatisfactory transport and internet services.

### 3.1. Socioeconomic Challenges

#### 3.1.1. Scarce Poverty Alleviation and Loan Schemes

Economic challenges, particularly in the current economic downturn, were emphasized as the barriers to MCH by the study participants, highlighting the need for poverty alleviation interventions and micro-loan schemes with no interest or low interest. Many participants confirmed that loan services were available through private providers and that cash transfers for poor families were, in theory, made by the government poverty alleviation (PA) programs, but they also described the problems therein. Loan providers charged prohibitive high-interest rates. In contrast, since interest on loans is not aligned with the values of the majority religion in the country, the expectation of the poor and underprivileged is to secure interest-free loans at reasonable terms of return that are culturally and religiously acceptable. Furthermore, the cash transfers from the government’s PA schemes were not easily accessible, or they were too small to make any difference in the lives of the poor. The following quote captures some of these issues:

“There are loans available from institutions like ‘Kashf’ and ‘Daman’ [referring to Non-governmental organizations], but the interest rates are very high. We take loans for very important things… like if we need money for our daughter’s marriage. We do not want to take loans or be in debt, we want employment opportunities and a regular income.”

[FGD 02 with women of reproductive years, 54-year-old widow].

“If Zakat was collected in an organized way and given to people who rightfully deserved it, poverty would cease to exist. These loan schemes have interests. Riba (usury) destroys societies and nations. No matter the need, we do not allow our daughters to take loans. Do you young people [referring to data collectors] remember the story of Abdul Qadir Jillani? He said that his mother was his biggest teacher. His mother would recite the Quran when she was pregnant, and this influenced him (Jillani) to become a pious person. If our women start engaging in usury, it will destroy our society. It is an illusion that loans based on interest can eradicate poverty or help a household.”

[IDI 01 with father-in-law, 72 years old].

“A lot of times I felt the need to apply for a loan… for example to buy my own house, but when you apply for loans they [the banks] ask for several documents. They ask for guarantees (land documents) to issue loans and we don’t have anything to pledge against the loan. There is no benefit of a government scheme like BISP, it is just an allowance but cannot help us to buy our own homes.”

[IDI 05 with a male household member, 27 years old].

The problems in the government poverty elimination programs are multifaceted. These programs are typically identified with the political party in government at the launch of these programs. When the governments change, the incoming political party dismantles the existing programs to rebrand them so that they are identified with their party. For instance, many people receiving the Ehsas Program card could no longer benefit from the same program due to a change in government in 2022. As a result, the governing party uses the rebranded programs in their election campaign, alienating the masses who are the opposition party supporters. Other problems with the PA programs, such as the Ehsaas Program, Benazir Income Support Program, and the Sehat Sahulat Program, include the politicization of red tapism in these programs. The loans are structured so that securing a loan is next to impossible for poor people due to collateral requirements that, by definition, poor people rarely can fulfill in the loan application. In addition, cash transfers are structured in a way that there are limited benefits of such transfers:

“I tried to get registered for the Benazir Income Support Programme (BISP), I tried to apply for the card but it was never processed (due to eligibility issues). We got a cash transfer from Ehsaas Program during COVID, but it was not repeated. As regards the Sehat Sahulat Program, we hear about it and watch news about how a health card has been given to everyone. This is not true, we don’t have one. I had to take my mother to an emergency [care facility] when she experienced pain. It turned out to be a [kidney] stone. There was no time to apply for the card. We asked people at the hospital, but no one helped us. These are just scams for publicity, we don’t have the facility of cash transfers or health cards for the poor.”

[IDI 04 with husband, 42 years old].

#### 3.1.2. Inadequate Food Supply and Safety

Most participants emphasized the exponential price-hikes for basic food items and their inability to afford a sufficient quantity of quality food, including basic food items such as pulses, wheat, fruits, vegetables, and milk. The unprecedented inflation, particularly in the price of food and groceries, has driven an estimated 37% of Pakistanis below the poverty line of USD 3.65 per day into a vicious cycle of food insecurity, malnutrition, and undernutrition [36]. Accordingly, study participants also confirmed that they ate only one or two meals a day, for the main course, due to the expense and rising prices of food in the last few years. One female participant described her household situation as:

“Food is now unaffordable and not of good quality either. We barely afford to eat one meal a day. The water needs to be boiled and there is little time for this, to be honest. My husband is a daily wage earner and with two young children (2 years and 3 years) there is no money for milk. My father-in-law is bedridden and a cancer patient. Everyone says to fix his diet and give him organic food. Where should we buy this from and where should we get the money?”

[IDI 04 with a female participant, 24 years old].

Health professionals’ observations of their communities corroborate the grim situation of food insecurity, the non-availability and affordability of healthy foods, and the worsening situation of food production, highlighted by the community participants. These health professionals underscored health problems such as anemia, stunted growth of children, low-calorie intake in pregnant women, and other related health issues facing women and children as a consequence of the inability of the masses to meet the basic food requirements. As members of the communities they serve, some public health professionals also linked food insecurity to the country’s economic instability, wage stagnation, and escalating food costs, considering them among the barriers to MCH:

“We have a lot of anemia patients or those reporting weakness. Also, there is a lack of calcium in women. Girls have issues of growth for which they need calcium. There is an overall lack of nutrients as the food quality is not good these days. All food is adulterated. Our job is to emphasize to them that taking medicines is not enough. They need to consume milk and fruits and vegetables. But there are issues of cost and access to organic food. In the past, people could afford to raise and own cows, but now due to rapid urbanization and the building of so many commercial plazas, there are no places to raise cows or grow fresh fruits and vegetables in the cities. If there is anemia, we ask them to consume more red meat but that option is not available as the cost of meat is very high. Some of these families only consume meat if it is given to them by wealthy families on the *Eid* festival or if they attend a wedding/funeral, where it may be served to guests. We need to give iron supplements, folic acid, and calcium supplements free to women and girls- we need to consist supplies from the center.”

[FGD 02 with BHU Team, doctor, and women of reproductive years, 38 years old].

#### 3.1.3. Unsatisfactory Public Sector Services (Schools and Health Services)

With the understanding that education, including health education, is an important influence on health and wellbeing, the narratives of the study participants overwhelmingly accentuated the inferior quality of the services and infrastructures of the public sector schools in their community. Not only were the schools described as unclean and overcrowded, with small rooms that did not have good lighting or ventilation and few and badly maintained toilets, but the teachers and administration were described as ‘rude’, ‘incompetent’, and ‘negligent’. Community participants confirmed that most students attending the public schools had to be supported with tuition otherwise they would not be able to complete the Matric or FSc exams (external board exams for grades 10 and 12 that are needed to apply for university). The paradoxical choices facing families who want quality education for children but cannot afford the reputed private schools with prohibitive fee structures were also described by some participants:

“Have you ever visited these public schools… the washrooms, the canteen, the rooms where children study. There is one washroom which is not clean and always smells. The canteen is dirty and not monitored. The classrooms are overcrowded and with bad lighting. The teachers and administration do not talk to us or listen to us. None of the children want to stay in school or like going to school. We do not have money for the private schools which I hear are better… and also they are too far away. Who would take them (the children)? I am sending my son for tuition, so that at least he passes the board exams, as the teachers do not cover the syllabus.”

[IDI 03, with Lady Health Worker, 42 years old].

“No, my children (two sons and one daughter) do not go to school because of the high fees of private school. The public schools also require money for other expenses, which we do not have. Also, schools require B-forms which we do not have (A birth certificate in Pakistan is known as a B-form. The document is used to register minors under the age of 18 years and is needed for seeking loans, school registration, hospital cards, traveling, etc.). My husband is a laborer and we live in a rented room. We barely make ends meet for rental payments and food.”

[FGD 05 with female participants, 45 years old].

A mother described the common health issues faced by children attending public schools in her community due to a lack of available safe drinking water:

“There is no filtered water in the public schools. There are water tanks with piped water, but they have no lids and the children are forced to drink that water. Of course, we give our children water bottles, but it is so hot most of the year, that they need to refill their bottles. This is why our children have constant stomach issues and miss their classes because of absenteeism due to frequent illness.”

[FGD 04 with female participants, 30 years old].

The participants chose to speak about the BHU services, though this question was not included in our semi-structured guide. They described problems related to the availability of medicines, resources, and health staff. A male community leader shared:

“Four years ago I talked to the doctor in charge at our local BHU and asked him for five things: I demanded that the BHU should be well stocked with medicine, the MBBS doctor should be on duty 24/7, there should be an ambulance available, there should be security outside the BHU, and a water filtration plant. The last is the most important, most of the health problems are related to unsafe water, and security is important as women find it unsafe to travel to and from the BHU alone. There was no response. This is the respect that they have for us. These buildings (BHU) are probably just to show the world and help politicians to win seats. They are not to help the poor women or other members of society who need proper health services.”

[IDI 02 with male community religious leader, 72 years old].

Another participant described their frustration about the lack of coordination between state structures, including the public health sector, and the local government offices, such as the waste management and water supply and sanitation department:

“We cannot understand the disconnect between the BHU and the government. The BHU doctor tells us to give our children safe water, dispose of our garbage, and keep the children from infectious diseases. Can you see this neighborhood? There is no one to collect the garbage and the drainage gutters are open. You can smell it too, even if you are blind.”

[FGD 06, women of reproductive years, 35 years old].

### 3.2. Environmental Challenges

#### 3.2.1. Poor Quality of Housing and Sanitation Services and Rent Burden

Nearly all participants confirmed that the communities they lived in had inadequate housing utilities, such as gas, water supply, and electricity. There were issues relating to a lack of supply, shortages, intermittent supplies, and the inferior quality of utilities provided at central locations such as the communal water filters. Study participants attributed some MCH issues to the inadequacy and inaccessibility of these utilities:

“This area does not have adequate gas and water supply from the government. There is also no supervision or consistent collection of garbage. There is a need for the people to follow up with the government, but they do not pay heed to the garbage disposal and have become accustomed to shortages in the supply of electricity, water, and gas.”

[FGD 01 with BHU team, female doctor, 45 years old].

“Gas shortages mean that we have very little cooking time. Our gas comes after 6 am and then stops at 8 pm. When it is available, there is very little supply and we manage to cook only one handi (one gravy-based meal) and rotis (bread) for the day. We do not have enough gas to boil water for drinking. This is probably the reason for the frequent stomach pain in our children. The LHWs warn us regularly, but what can we do? We can either boil water for drinking or cook food to eat.”

[IDI 06 with a woman of reproductive years, 31 years old].

Study participants also highlighted environmental health problems emanating from poor quality of housing, housing materials, and excessive rent burdens coupled with the unjust practices of landlords. There was frustration that, despite high rent costs, the landlords were not providing adequate maintenance of the rental houses. At the same time, the tenants faced health hazards from poor maintenance, causing leaking roofs, damaged walls and peeling-off paints, damp and moldy houses, overcrowded neighborhoods and houses, and a lack of privacy due to such crowding. Although the participants may not have known the scientific mechanisms linking these environmental health hazards, they attributed some respiratory and skin conditions to those poor environmental conditions:

“Some of the houses in the community have concrete roofs, but others are not in good condition or made of adequate material. Leaking is common and winter and rainy season can especially be difficult. Let’s say, there are 50 houses and 20 of them are not safe to live in. Many people ask us for *Masjid chanda* (charity donated to the mosque) to repair their houses, but we do not have money for this—as we can only manage food in such a situation.”

[IDI 01 with male community religious leader, 62 years old].

“Our house is old and damp. We cannot keep the cockroaches or the termite away. My father-in-law and the LHW say that this is why my little one (infant son) has a chronic cough. But, there is no money to fill the cracks (in the walls and roof), and removing the dampness from the house is not in my control. Whenever it rains the water collects in the streets. The sewerage system is not satisfactory at all. Even though the sewerage system is closed, sometimes the gutters get blocked and dirty sewage water leaks into the streets. Mosquitoes gather over the stagnant water making it filthier. For months the streets remain this way and nobody comes to drain them. When the streets need to be cleaned the residents of the area collect funds themselves and call services to clean the water otherwise no one pays attention to the dirty conditions.”

[IDI 05 with women of reproductive years, 32 years old].

The lack of home ownership and the burden of rental amounts negatively impacted families’ ability to purchase other life necessities such as food and clothing. The participants perceived high rent amounts to create a zero-sum situation with the purchase of healthy and sufficient calories:

“We pay PKR 16,000 a month for rent. Both my son and daughter work. It’s very hard. My daughter earns PKR 10,000 a month by tutoring kids and my son is a daily wage earner. He earns around PKR 500–700 per day. High rent payments mean that we only eat once a day.”

[FGD 02 with women of reproductive years, 50-year-old widow].

“Rent is very high in this area and families are suffering. The women regularly complain that there is no money for other necessities, such as milk for young children or clothes in winter as money has to be saved for high rent payments. There needs to be a ceiling for rents for poor families.”

[FGD 01 with BHU team, Lady Health Worker, 38 years old].

#### 3.2.2. Lack of Safety and Security

The majority of participants raised neighborhood safety concerns and resulting stress, anxiety, and fear. The lack of security in their community also meant that many women adopted preventive strategies such as not traveling alone, not traveling after dusk, and not venturing far from their homes to remain safe. An LHW shared:

“It’s not safe at all. We never send our young girls alone outside unless there’s someone with them. During the day, I’m not afraid but when it starts getting dark I am scared. Somebody snatched another LHWs purse once in the evening. She was not far from her home, which was the worst part. We do not report theft and harassment to the police as they do not respond or help.”

[IDI 01 with LHWs, 38 years old].

“One has to save oneself on one’s own, it’s that simple. No one is going to save you. There is no security or law. The situation these days has gotten worse; anyone can mug you at gunpoint. We have heard of the kidnapping of girls also. This is why we do not let our daughters go anywhere alone or even play outside the home.”

[IDI 03, with women of reproductive years, 38 years old].

#### 3.2.3. Unsatisfactory Transportation and Internet Services

Though transport services were available, the major challenges described by participants included the distance and the inability of women to travel alone, both due to lack of permission from family and also safety concerns. Internet was only available if people were willing to pay for it, and it was mostly used by male household members and not women. The participants felt that the lack of internet access limited their ability to access health information and stay informed about health hazards. Some participants also referred to the possibilities of telehealth consulting, but others thought that telehealth had its limitations:

“I have a wife and a 4-month-old son. The terminal is close by, but we do not allow our women to use public transport. It is not safe or allowed in our family. We take them where they need to go. Yes, I need internet for my studies and work, but I pay for the Jazz internet card used on my phone. My wife does not need or use the internet.”

[IDI 02 with a household member; a 20-year-old husband].

“If we (women in the community and relatives who are pregnant) have complications or need to discuss some unusual symptom during pregnancy, we ask our local Dai… she also manages the delivery. Thank God we have them. It is not good to depend on doctors who will never visit our house or do not know our families. How can we depend on online health services or the internet for the health and delivery of babies, it is ridiculous. This is something that will not last. You all (youth and those using the internet) should plan lives without being so dependent on electricity and internet… and phones.”

[IDI 01 with mother-in-law and 60-year-old husband].

## 4. Discussion

This study has summarized findings from community needs assessments to understand what socioeconomic and environmental challenges may be influencing the health of mothers, children, and community members living in the urban slums of Pakistan. Our findings suggest that inadequate housing, inferior sanitation, inadequate disposal of wastewater and solid waste due to inadequate public services, inadequate supply of utilities, unsafe drinking water, and high rents in the communities were substantial challenges to the behavioral and physical health of women and children. Other local research studies have also suggested that urban cities in Pakistan have inferior housing policies and large urban slum zones [37,38], which can contribute to health hazards, including both mental and physical issues [39]. Our findings show that basic food items are expensive and inaccessible in most disadvantaged communities. There are also issues of adulterated food and a lack of good-quality food items. As a result, families suffer from undernutrition and eat fewer meals a day. Other local research studies also suggest that rising food prices [40] and the circulation of adulterated food in urban cities [41,42] can lead to food insecurity and undernutrition in poor families [43].

The current study found that public sector school services are low in quality but not completely free, preventing the poorest of the poor from obtaining a basic education for their children. Among some low-income families, parents cannot afford to procure quality education for all children and are forced to choose who among their children will receive private school education, resulting in structural gender discrimination. Studies have shown that the inferior quality of schooling and teaching leads to issues surrounding the mental and physical health of mothers and children [44,45,46]. Our community needs assessment also reveals that the majority of participants do not feel safe in their community and lack security and surveillance. This also has implications for mothers making choices not to visit healthcare centers due to fear for their safety. Most of the community members are fearful that they may be robbed at any time or that females may face harassment or kidnapping. Research from South Asia confirms that this region is an unsafe place for females [47,48] and that human trafficking is a serious safety and health issue for females [49,50].

The findings reveal that impoverished communities have little support from the government for poverty alleviation or small business mobilization. All the participants reported that no government schemes were available for loans, and, though private providers were disbursing loans, the installment amounts were high. More importantly, community members, especially elders, were not in favor of loans, which they associated with interest and considered un-Islamic [51]. Other research confirms that small loans with high-interest rates trap the poor in endless debt cycles and contribute to stress and anxiety [52,53].

Our findings confirm that, though cash transfer schemes (like BISP and Ehsaas Program) and health insurance schemes (Sehat Sahulat Program) by the government are advertised and known by poor community members, they had difficulty availing them and becoming beneficiaries. Other research confirms that the majority of the poor and deserving in Pakistan have been unable to avail themselves of the BISP [54], the Ehsaas Program [55], or the Sehat Sahulat Program [56] due to low outreach and the mismanagement of limited funds.

Our findings confirm that public transport is available, but there are issues of access, distance, and safety for women. Furthermore, women are prevented from traveling alone without male guardians due to safety and family permission issues. Other local literature confirms that women in the urban zones need pink or women-only transport services which are of good quality and accessible in terms of cost and distance [57,58], especially for women of reproductive years who need to travel alone for the health needs of themselves and their children [59]. Our findings also reveal that there are no public WiFi or internet access schemes provided by the state and that most men in the community use the internet and pay for it out-of-pocket. Local research confirms that internet access is a major barrier to not just health access [60], but also to work and education, for the poor and middle classes [61]. Ultimately, supporting poor women for e-health and other opportunities and information through the Internet will require state support for free WiFi, smartphone provision, and awareness sessions with families about the importance of women having access to the Internet [62].

Finally, our study found that BHU services in urban communities are perceived as inadequate and non-dependable by community members. There were complaints of a lack of supplies and medicine, absent healthcare staff, and a lack of space. Grievances were also shared about the curt behavior of healthcare staff and the lack of coordination between the BHU teams and other state services in the community, for example, departments managing waste, water, and sanitation. International literature confirms that primary healthcare must have efficient services and resources to build trust and uptake in the community [63], and, for this, the primary healthcare team and local governance must work closely with each other [64]. The health and well-being of the community members can suffer on multiple levels when there is a lack of coordination between the primary health sector and local governing bodies, including the exacerbation of infectious disease burden [65], inadequate management of mental health services [66], deficient safety and security [67], and insufficient support for poverty alleviation [68].

Innovative and robust as it is, the current study must be interpreted within the context of its limitations. The study participants came from the disadvantaged and underserved areas of Lahore city, the second largest city in the country, with a population of 11.1 million and a population density of 6300 people per square kilometer. Consequently, the challenges to MCH featured in this research may be different from those facing women, children, and families across smaller cities, towns, and villages, particularly because the focus is on the community characteristics and the SDoH therein. Secondly, although the research team considered the size of study participants sufficient to reach saturation of ideas, there was no scientific test of such saturation. Regardless of these limitations, this study makes a significant addition to the existing body of research evidence for health policy.

## 5. Conclusions

Based on our study findings in Lahore City, Pakistan, we conclude that maternal and child health cannot be considered an individual’s responsibility. Instead, systems-level improvements in the social determinants of maternal and child health are essential. Firstly, investments and resource commitments at the policy level are essential for improvements in communities to eliminate the environmental conditions associated with poor household maintenance and lack of access to utilities in disadvantaged neighborhoods. There is a critical need for applying health equity and Health in All Policies frameworks for improving families’ living conditions, including access to safe water, affordable and safe disposal of wastewater and solid waste, and affordable yet reasonable quality housing in poor neighborhoods. There is also a need for regulating private landlords for imposing fair rent ceilings for poor families and securing healthy housing for the poor. Secondly, equitable and inclusive access should be assured to good quality public schools, public transportation systems, and the Internet. Most importantly, solutions for safety, gated communities, street surveillance through cameras, and swift accountability against perpetrators must be introduced urgently, otherwise even good quality state services will not be accessed due to issues of safety. Thirdly, BHUs must be adequately staffed, budgeted, and trained in evidence-based public health to deliver MCH wrap-around and direct clinical services. The BHU staff should also be trained in the efficient administration and delivery of MCH services through resource-sharing, collaboration in implementing MCH solutions, and ongoing communications with all public health stakeholders. All state-managed local government offices must be accountable to a third-party accrediting body to assure performance management, adequate service administration, evidence-based decision making, quality improvement, accountability, resource sharing, and other standards established by the accrediting body. Community satisfaction surveys must also be used to incentivize and renew the contracts of local government officers. Lastly, system-level (macro) solutions need to be developed to ensure equitable access to SDoH, including efficient and transparent poverty elimination schemes, health insurance schemes, and state-regulated, interest-free, or low-interest loan schemes with capping on installments and reasonable requirements of loan collaterals. Substantial investment in interest-free loan services for small business mobilization, marriages, and higher education will also move the health–equity needle in the right direction.

## Figures and Tables

**Table 1:** Summary of IDIs and FGDs conducted with participants for community needs assessment.

Type of Study Participant	IDI	FGD
BHU Team		04
LHW	04	
Women of reproductive years	06	06
Male household members	05	
Mothers-in-law	03	
Religious leaders	04	
Total	22	10

Abbreviations: IDI, in-depth interviews; FGDs, focus group discussions; BHU, basic health units; LHW, Lady Health Worker.

## Data Availability

The datasets analyzed during the current study are available from the corresponding author upon reasonable request.

## Supplementary Materials

The following supporting information can be downloaded at: https://www.mdpi.com/article/10.3390/healthcare11152224/s1, File S1: Community Needs Assessment Interview Guide; File S2: Data Collection Location points in Lahore City, Pakistan.File S3: Pictures of data collection sites showing the living conditions of the community.
