# Supplementary material for: Characterizing Social Determinants of Maternal and Child Health: A Qualitative Community Health Needs Assessment in Underserved Areas
Source: Healthcare (Basel). 2023 Aug 7;11(15):2224. doi: 10.3390/healthcare11152224 (PMC10418942; doi:10.3390/healthcare11152224)
Supplement: Supplementary file 1 [file healthcare-11-02224-s001.zip › healthcare-2422209-supplementary.pdf]

File S1: Community Needs Assessment Interview Guide

Informed Cover letter and Consent for Participation in Study

Thank you for your valuable time.
We request you to participate in a research project that aims to understand the community liced experiences and needs with respect to ….
This study will help to improve health and social service delivery in your community and also improve health outcomes community members. At a broader level this study's findings will enable us to communicate with the government and policy-makers for improvement in your health, social protection floor and the overall development of your community.
All information collected during this study will be confidential. No names will be mentioned in publications and all information will be stored anonymously with the lead researcher.
If you agree to participate we will conduct 2-3 FGDs and IDIs with you and your fellow community members over the course of the next 2-3 weeks.
We hope you are willing to support this project and that you agree to part of this study.

Sincerely
Dr. Sara Rizvi Jafree,
Lead Researcher, Assistant Professor, Forman Christian College\ University
E-mail: sarajafree@fccollege.edu.pk
Cell: 0300 400 5740

مطلع کور لیٹر اور مطالعہ میں شرکت کے لیے رضامندی۔

اپکے قیمتی وقت کا شکریہ۔

یہ مطالعہ آپ کی کمیونٹی میں صحت اور سماجی خدمات کی فراہمی کو بہتر بنانے میں مدد کرے گا اور کمیونٹی کے ممبران کی صحت کے نتائج کو بھی بہتر بنائے گا۔ وسیع تر سطح پر اس مطالعے کے نتائج ہمیں آپ کی صحت، سماجی تحفظ کی منزل اور آپ کی کمیونٹی کی مجموعی ترقی میں بہتری کے لیے حکومت اور پالیسی سازوں کے ساتھ بات چیت کرنے کے قابل بنائیں گے۔
اس مطالعہ کے دوران جمع کی گئی تمام معلومات خفیہ ہوں گی۔ اشاعتوں میں کسی نام کا ذکر نہیں کیا جائے گا اور تمام معلومات مرکزی محقق کے پاس گمنام طور پر محفوظ کی جائیں گی۔
اگر آپ شرکت کرنے پر اتفاق کرتے ہیں تو ہم اگلے 2-3 ہفتوں کے دوران آپ اور آپ کے ساتھی کمیونٹی کے اراکین کے ساتھ ملاقات کریں گے۔
ہم امید کرتے ہیں کہ آپ اس پروجیکٹ کی حمایت کرنے کے لیے تیار ہوں گے اور یہ کہ آپ اس مطالعہ کے حصے سے اتفاق کرتے ہیں۔
مخلص
ڈاکٹر سارہ رضوی جعفری،
لیڈ ریسرچر، اسسٹنٹ پروفیسر، فارمن کرسچن کالج یونیورسٹی

Interview Participants:

- LHWs and LHSs= Mam Khadija
- Community elders and religious leaders= Sara
- Women of reproductive years + women above 49 years = Anam and Ambreen
- Local government officials = Humna and Mam Rubab

A. Housing and sanitation

i. Are you happy with the housing quality in your community with regard to waste disposal, electricity, and gas?

کیا آپ کچرے کو ٹھکانے لگانے، بجلی اور گیس کے حوالے سے اپنی کمیونٹی میں رہائش کے معیار سے خوش ہیں؟

ii. Are you happy with the affordability of rent in your community?

کیا آپ اپنی کمیونٹی میں کرایہ کی سطح سے خوش ہیں؟

iii. Are you happy with the livability aspect of your housing, specifically 1. Is it in good repair, 2. Is it big enough, 3. Is it free of hazardous material and unsafe pollutants, 4. Is it a safe neighborhood overall, 5. Is there access to groceries, public transport and health center?

کیا آپ اپنی رہائش کے رہنے کے پہلو سے خوش ہیں، خاص طور پر 1. کیا یہ اچھی طرح سے مرمت ہے، 2. کیا یہ کافی بڑا ہے، 3. کیا یہ خطرناک مواد اور غیر محفوظ آلودگیوں سے پاک ہے، 4. کیا یہ مجموعی طور پر ایک محفوظ پڑوس ہے، 5. کیا گروسری، پبلک ٹرانسپورٹ اور ہیلتھ سینٹر تک رسائی ہے؟

iv. Do you feel safe living in the house during uncertain weather and extreme conditions? (AM)

کیا آپ غیر یقینی موسم اور انتہائی حالات میں گھر میں رہنا محفوظ محسوس کرتے ہیں؟

(Source: Community Tool Box- https://ctb.ku.edu/en/table-of-contents/implement/physical-social-environment/housing-quality/main)

B. Water and food security

i. Are you happy with the access to safe drinking water in your community?

کیا آپ اپنی کمیونٹی میں پینے کے صاف پانی تک رسائی سے خوش ہیں؟

ii. Are you happy with the sufficient quantity of running water that reaches your homes, for washing and bathing?

جو پانی آپ کے گھروں تک، نہانے دھونےکے لیے پہنچتا ہےاس کی مقدار سے خوش ہیں ؟

iii. Are you satisfied with access to food and all the varieties of food available in your neighborhood (e.g. milk, meat, vegetables, fruit, lentils, wheat)?

کیا آپ خوراک تک رسائی اور اپنے پڑوس میں دستیاب تمام اقسام کے کھانے (مثلاً دودھ، گوشت، سبزیاں، پھل، دال، گندم) سے مطمئن ہیں؟

iv. Are you satisfied with the cost of different food types available in your neighborhood?

کیا آپ اپنے پڑوس میں دستیاب کھانے کی مختلف اقسام کی قیمتوں سے مطمئن ہیں؟

iv. Are you satisfied with the quality of different food types available in your neighborhood?

کیا آپ اپنے پڑوس میں دستیاب کھانے کی مختلف اقسام کے معیار سے مطمئن ہیں؟

C. Schooling and education services

i. Are you happy with the availability of primary and secondary schools for boys and girls in your community?

کیا آپ اپنی کمیونٹی میں لڑکوں اور لڑکیوں کے لیے پرائمری اور سیکنڈری اسکولوں کی دستیابی سے خوش ہیں؟

ii. Are you happy with the quality of primary and secondary teaching and school infrastructure & administration for boys and girls in your community?

کیا آپ اپنی کمیونٹی میں لڑکوں اور لڑکیوں کے لیے پرائمری اور سیکنڈری تعلیم اور اسکول کے بنیادی ڈھانچے اور انتظامیہ کے معیار سے خوش ہیں؟

iii. Do you have access to public and private schools within your community? (HA)

کیا آپ کو اپنی کمیونٹی میں سرکاری اور نجی اسکولوں تک رسائی حاصل ہے؟

iv. Do you feel girls and boys have the equal access to education in your community?

کیا آپ کو لگتا ہے کہ آپ کی کمیونٹی میں لڑکیوں اور لڑکوں کو تعلیم تک یکساں رسائی حاصل ہے؟

v. Do your children feel safe while attending school?

کیا آپ کے بچے اسکول جانے کے دوران محفوظ محسوس کرتے ہیں؟

vi. Has your child ever been bullied by other fellow students/friends?

کیا آپ کے بچے کو کبھی دوسرے ساتھی طلباء/دوستوں کی طرف سے تنگ کیا گیا ہے؟

vii. Do schools in your community have space for recreation within the compound of the school?

کیا آپ کی کمیونٹی کے اسکولوں کے پاس اسکول کے احاطے میں تفریح کے لیے جگہ ہے؟

viii. Do schools in your community have access to a source of safe water within/near to the school compound?

کیا آپ کی کمیونٹی کے اسکولوں کو اسکول کے احاطے کے اندر/قریب محفوظ پانی کے ذرائع تک رسائی حاصل ہے؟

ix. Are you happy with the availability of tuitions and training centers in your community?

کیا آپ اپنی کمیونٹی میں ٹیوشن اور تربیتی مراکز کی دستیابی سے خوش ہیں؟

x. Are you happy with the school transport?

کیا آپ سکول ٹرانسپورٹ سے خوش ہیں؟

xi. Are you happy with access to universities in your community or the transport to them??

کیا آپ اپنی کمیونٹی کی یونیورسٹیوں تک رسائی یا ان تک ٹرانسپورٹ سے خوش ہیں؟

xii. Are you happy with job prospects for both males and females in your community after graduation?

کیا آپ گریجویشن کے بعد اپنی کمیونٹی میں مردوں اور عورتوں دونوں کے لیے ملازمت کے امکانات سے خوش ہیں؟

D. Safety and security

i. Are you happy with the safety and security in your neighbourhood?

کیا آپ اپنے پڑوس میں تحفظ اور سلامتی سے خوش ہیں؟

ii. Are you happy with the police and other security agents deployed in your community?

کیا آپ اپنی کمیونٹی میں تعینات پولیس اور دیگر سیکورٹی ایجنٹوں سے خوش ہیں؟

iii. Are you happy with the acceptance level of people from different areas (ethnicity, religious groups, cultural background) when they come to reside or visit your community?

کیا آپ مختلف علاقوں (نسل، مذہبی گروہ، ثقافتی پس منظر) کے لوگوں کی قبولیت کی سطح سے خوش ہیں جب وہ آپ کی کمیونٹی میں رہنے یا ملنے آتے ہیں؟

iv. Are you aware of any particular group that suffers from lower safety in your community?

کیا آپ کسی ایسے مخصوص گروہ سے واقف ہیں جو آپ کی کمیونٹی میں کم حفاظت کا شکار ہے؟

v. Have you or any of your family members ever felt threatened by someone in your - neighborhood/community?

کیا آپ یا آپ کے خاندان کے کسی فرد کو کبھی آپ کے محلے/کمیونٹی میں کسی سے خطرہ محسوس ہوا ہے؟

vi. Are you aware of who you should complain to in case of any safety issue arises?

کیا آپ اس بات سے واقف ہیں کہ حفاظتی مسئلہ پیدا ہونے کی صورت میں آپ کو کس سے شکایت کرنی چاہئے؟

vii. Are you equipped for countering any threatening situation in your neighbourhood?

کیا آپ اپنے پڑوس میں کسی بھی خطرناک صورتحال کا مقابلہ کرنے کے لیے تیار ہیں؟

viii. Are you happy with safety and security situation while commuting and using public transport?

کیا آپ پبلک ٹرانسپورٹ کے سفر اور استعمال کے دوران حفاظت اور سلامتی کی صورتحال سے خوش ہیں؟

E. Availability of loan, entrepreneurial opportunities and poverty schemes

i. Are you happy with availability of loans (for health, small business, other things) in your community? *(Prompt: What kind of loans would you be interested in which are not available yet?)*

کیا آپ اپنی کمیونٹی میں قرضوں (صحت، چھوٹے کاروبار، دیگر چیزوں کے لیے) کی دستیابی سے خوش ہیں؟ (پرامپٹ: آپ کس قسم کے قرضوں میں دلچسپی لیں گے جو ابھی دستیاب نہیں ہیں؟)

ii. Are you happy with opportunities for starting small entrepreneurial and business activities in your community? *(Prompt: If not, what are the problems you face?)*

کیا آپ اپنی کمیونٹی میں چھوٹی کاروباری اور کاروباری سرگرمیاں شروع کرنے کے مواقع سے خوش ہیں؟ (پرامپٹ: اگر نہیں، تو آپ کو . کن مسائل کا سامنا ہے؟)

iii. Are you happy with availability of poverty alleviation schemes, savings schemes and insurance schemes in your community?

کیا آپ اپنی کمیونٹی میں غربت کے خاتمے کی اسکیموں، بچت اسکیموں اور انشورنس اسکیموں کی دستیابی سے خوش ہیں؟

iv. Are you happy with access and service of government offices (for different paper work, registration, and applications) in your community?

کیا آپ اپنی کمیونٹی میں سرکاری دفاتر (مختلف کاغذی کام، رجسٹریشن اور درخواستوں کے لیے) تک رسائی اور خدمات سے خوش ہیں؟

v. Are you happy with access and service of banks (for different paper work, registration, and applications) in your community?

کیا آپ اپنی کمیونٹی میں بینکوں تک رسائی اور خدمات (مختلف کاغذی کام، رجسٹریشن اور درخواستوں کے لیے) سے خوش ہیں؟

vi. Are you happy with availability of opportunities to sell your products and services in community and beyond?

کیا آپ کمیونٹی اور اس سے باہر اپنی مصنوعات اور خدمات فروخت کرنے کے مواقع کی دستیابی سے خوش ہیں؟

vii. Are you happy with the access to information for entrepreneurial activities and updates about capacity building opportunities (e.g. new micro credit schemes, new tax laws for small businesses) in your community (e.g. TV, radio, wifi and internet).

کیا آپ اپنی کمیونٹی میں کاروباری سرگرمیوں کے لئے معلومات اور صلاحیت سازی کے مواقع (مثال کے طور پر نئی مائیکرو کریڈٹ اسکیمیں، چھوٹے کاروباروں کے لئے نئے ٹیکس قوانین) کے بارے میں رسائی سے خوش ہیں.

F. Transport Services

i. Does your community have access to local transportation?

کیا آپ کی کمیونٹی کو مقامی نقل و حمل تک رسائی حاصل ہے؟

ii. Does everyone in the community have equal access to these transport services?

کیا کمیونٹی کے ہر فرد کو ان ٹرانسپورٹ سروسز تک مساوی رسائی حاصل ہے؟

iii. How often will you be able to find transportation in case of an emergency?

ایمرجنسی کی صورت میں آپ کتنی بار آسانی سے سواری تلاش کر سکیں گے؟

iv. Are you able to obtain transportation during business hours when you need to go to the nearest town/village?

کیا آپ کاروباری اوقات کے دوران جب آپ کو قریبی شہر/گاؤں جانے کی ضرورت ہو تو آپ آسانی سے نقل و حمل کرنے کے قابل ہیں ؟

v. Is there a time that you were not able to go somewhere due to lack of availability of transportation services?

کیا کوئی ایسا وقت ہے جب آپ نقل و حمل کی سہولیات کی عدم دستیابی کی وجہ سے کہیں نہیں جا سکے تھے؟

vi. Have you ever felt harassed by other passengers during your travel on public transportation?

کیا آپ نے کبھی عوامی نقل و حمل پر اپنے سفر کے دوران دوسرے مسافروں کی طرف سے ہراساں کیا گیا ہے؟

vii. Are you allowed to travel alone?

کیا آپ کو اکیلے سفر کرنے کی اجازت ہے؟

viii. If no, who are you allowed to travel with?

اگر نہیں، تو آپ کو کس کے ساتھ سفر کرنے کی اجازت ہے؟

File S2: Data Collection Location points in Lahore City, Pakistan

#	Tehsil*	Area	Address	Google Map link
1	Cantt	Bhasin	JGV4+46P, Bhasin Lahore, Punjab	https://goo.gl/maps/3smdpq87ac4KKCC3A
2	Raiwind	Shahpur, Alama Iqbal Town	F653+P23, Shahpur Kanjra, Lahore, Punjab	https://goo.gl/maps/cZtsyVJXJiCgBXsc8
3	Raiwind	Munir Garden	F6QM+GXP, Munir Garden Thoker Niaz Baig, Lahore, Punjab	https://goo.gl/maps/fjmWM3wpueSN2sEu8
4	Iqbal Tehsil	Township Block 2, Sector C	C7QW+FQ8, Township Block 2 Twp Sector C 2 Lahore, Punjab 54600	https://goo.gl/maps/zoSToUt7Zo6W1NUf7
5	Cantt	H.B.F.C Society	B 16, Block B H.B.F.C Society, لاہور, Lahore, پنجاب	https://goo.gl/maps/cK4zF3cGT7hkhSZ89
6	Raiwind	Sundar Sharif, Raiwind Road	942H+R7X, Sundar Raiwind Rd, Sundar Sharif, Sundar, Lahore, Punjab	https://goo.gl/maps/ttRg89sbuxN2Dikg7
7	Cantt	Jallo Park	HFGQ+7MW, Unnamed Road, Jallo Village لاہور, Lahore, پنجاب	https://goo.gl/maps/s2xz4hQ4cV8cPHGM7

Notes:

* Lahore district is divided in to five Tehsils (administrative areas) including Lahore Cantonment (Lahore Cantt), Lahore City Tehsil, Model Town Tehsil, Raiwind Tehsil and Shalimar Tehsil https://lahore.punjab.gov.pk/constituencies. According to data available on Primary and Secondary Health Care Department Punjab, the BHUs of Lahore district are located in Tehsils of Lahore Cantt (18 BHUs) and Raiwind (18 BHUs) and only 1 BHU is under Lahore City Tehsil https://pshealthpunjab.gov.pk/Home/BHU

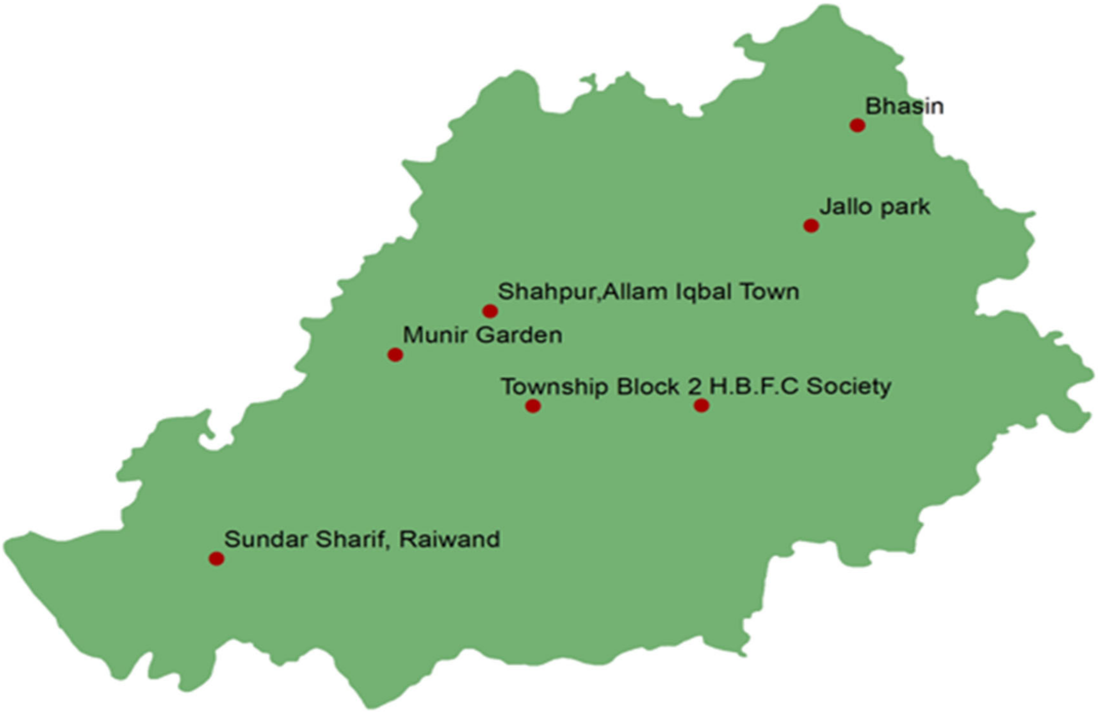

Figure 1: Underserved areas in Lahore where community needs assessment was conducted

File S3: Pictures of data collection sites showing the living conditions of the community

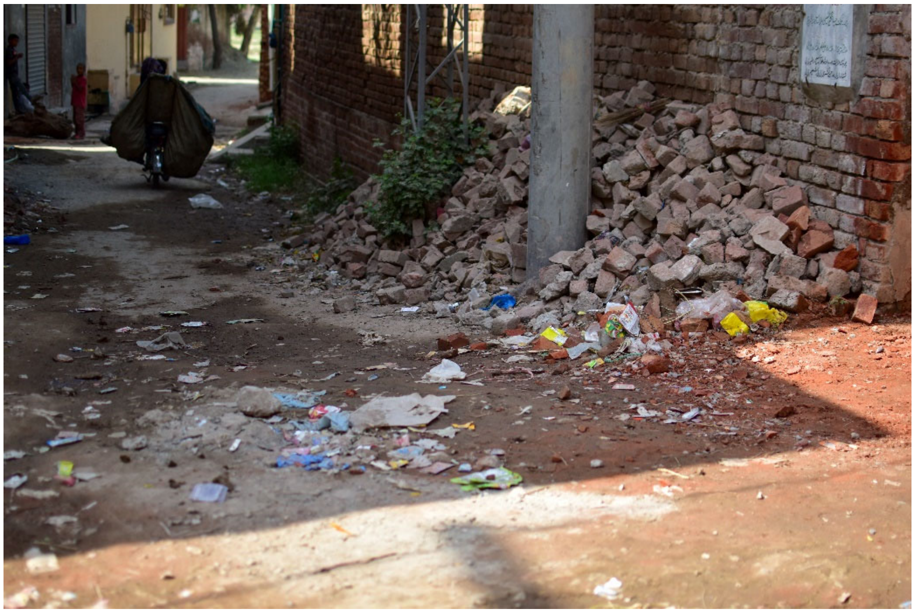

Image 2: Building obstruction on public streets and the state of garbage disposal; community images as at February 2023.

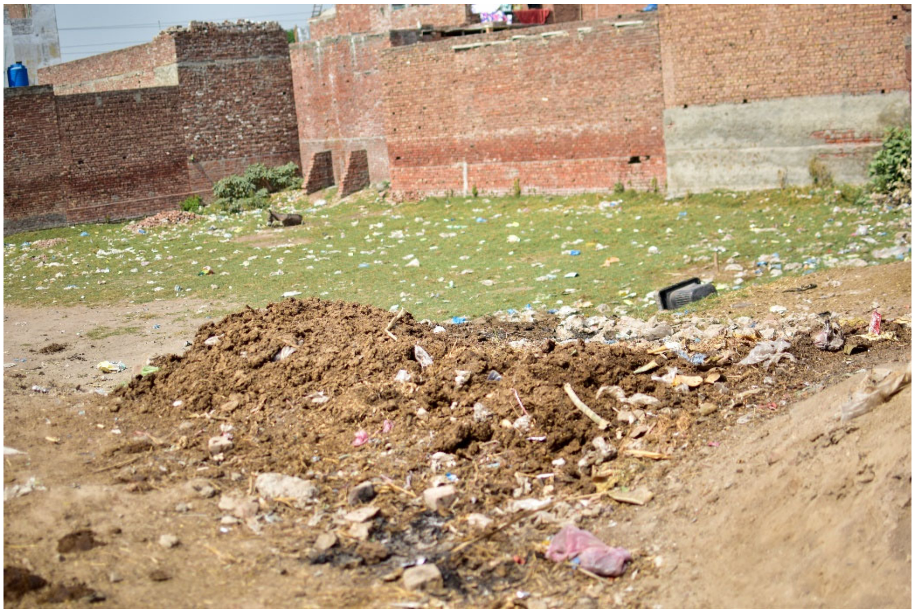

Image 2: Unmonitored garbage dumps in empty plots of the community. This space is also used in the night for setting fire to garbage by individual families; images as at February 2023.

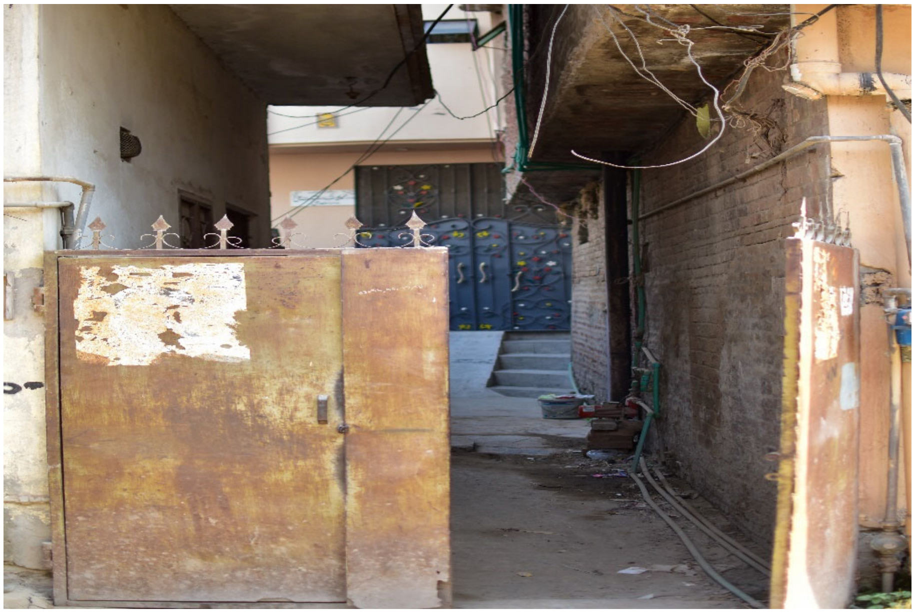

Image 3: Illegal construction of houses without adequate spacing and privacy for residences; images as at February 2023.

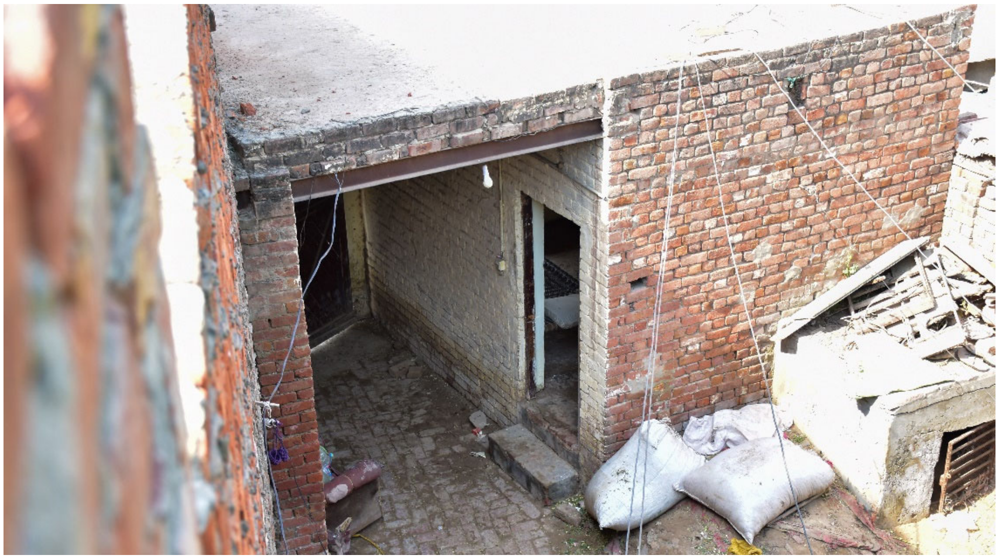

Image 4: One unit house of two rooms, with open spaces and unfinished walls, which face flooding and weather exposure; images as at February 2023.

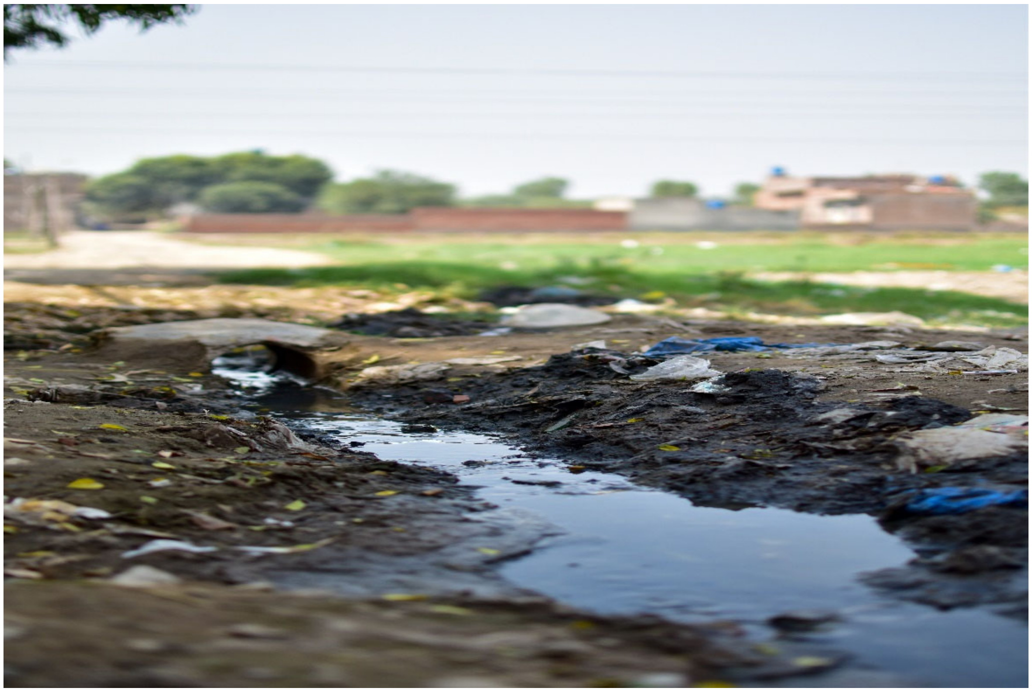

Image 5: Open drains and open gutters in the community; images as at February 2023.
